# Cell–cell fusion of mesenchymal cells with distinct differentiations triggers genomic and transcriptomic remodelling toward tumour aggressiveness

**DOI:** 10.1038/s41598-020-78502-z

**Published:** 2020-12-10

**Authors:** Lucile Delespaul, Caroline Gélabert, Tom Lesluyes, Sophie Le Guellec, Gaëlle Pérot, Laura Leroy, Jessica Baud, Candice Merle, Lydia Lartigue, Frédéric Chibon

**Affiliations:** 1 INSERM U1037, Cancer Research Centre in Toulouse (CRCT), 31037 Toulouse, France; 2 INSERM U1218, 229 cours de l’Argonne, 33076 Bordeaux, France; 3 University of Bordeaux, 146 rue Léo Saignat, 33000 Bordeaux, France; 4 IUCT-Oncopole, Institut Claudius Régaud, 31000 Toulouse, France; 5 Department of Pathology, Institut Claudius Régaud, IUCT-Oncopole, 31000 Toulouse, France; 6 University of Toulouse 3, Paul Sabatier, 118 route de Narbonne, 31062 Toulouse Cedex 9, France; 7 Present Address: Sarcoma Molecular Pathology Team, The Institute of Cancer Research, 15 Cotswold Road, Sutton, SM2 5NG UK; 8 Present Address: Science for Life Laboratory, Department of Medical Biochemistry and Microbiology, Biomedical Center, Uppsala University, Box 582, 751 23 Uppsala, Sweden; 9 Present Address: Cancer Genomics Group, The Francis Crick Institute, 1 Midland Road, London, NW1 1AT UK; 10 Present Address: ONCOSARC Team, Department of Biopathology and INSERM U1037, CRCT-IUCT-O, 2 Avenue Hubert Curien, CS 53717, 31037 Toulouse Cedex 1, France

**Keywords:** Cancer, Cancer genetics, Cancer genomics, Cancer models, Sarcoma, Tumour heterogeneity, Genetics, Cytogenetics, Genetic hybridization, Genome, Genomic instability, Genomics

## Abstract

Cell–cell fusion is a physiological process that is hijacked during oncogenesis and promotes tumour evolution. The main known impact of cell fusion is to promote the formation of metastatic hybrid cells following fusion between mobile leucocytes and proliferating tumour cells. We show here that cell fusion between immortalized myoblasts and transformed fibroblasts, through genomic instability and expression of a specific transcriptomic profile, leads to emergence of hybrid cells acquiring dissemination properties. This is associated with acquisition of clonogenic ability by fused cells. In addition, by inheriting parental properties, hybrid tumours were found to mimic the histological characteristics of a specific histotype of sarcomas: undifferentiated pleomorphic sarcomas with incomplete muscular differentiation. This finding suggests that cell fusion, as macroevolution event, favours specific sarcoma development according to the differentiation lineage of parent cells.

## Introduction

Cell–cell fusion is a physiological mechanism leading to the formation of hybrids through the union of two or more cells^1^. This mechanism is a fundamental and highly regulated process in mammals and is involved in fecundation, placentation, muscle development and osteoclast differentiation^1,2^. In addition, it is also involved in the homeostasis and contributes to tissue regeneration in liver, brain, muscle and lung^3^.

Cell–cell fusion is not limited to a normal physiological process^4,5^. Both normal and cancerous cells can hijack this physiological process to promote malignancies and to contribute to tumour evolution^6–12^. The direct consequence of cell–cell fusion is the combination in the same cell entity of the genomic and phenotypic properties of each parental cell, leading to the inheritance of parental traits in the hybrid^5^. This transmission process has been widely described, notably in the fusion between cancer cells and leucocytes, where hybrid cells take advantage of the properties of the parents by inheriting both tumoral proliferation and leucocyte mobility properties, hence promoting metastatic dissemination^6,7,13–15^. Another consequence of cell–cell fusion is the acquisition of new properties which can be explained by the genomic and transcriptomic reshuffling that occurs in hybrid following a cell–cell fusion event^16^. Moreover, some studies have demonstrated that cell fusion can promote tumour initiation or participate in tumour relapse with the acquisition of drug resistance^6,17–20^. Cell–cell fusion therefore contributes to genome doubling and reshuffling, leading to emergence of hybrids with a unique behaviour pattern. Mechanisms as *chromothripsis* or cytokinesis errors produce genome remodelling in a single event as well, a leap during tumour evolution called macroevolutionary event. However, cell fusion is the only macroevolutionary event promoting horizontal genetic transmission by merging distinct genomes. Consequently, cell fusion is an atypical process participating in tumour evolution.

To understand the role of cell–cell fusion in tumour evolution, especially during sarcomagenesis, we previously analysed spontaneous hybrids from immortalized i.e. non-transformed fibroblasts. We demonstrated that fused cells acquire new genomic alterations associated with the capacity for tumour development, proving that cell fusion can be involved at the initial tumour development step^20^. More recently, we showed that fusion between immortalized and transformed fibroblasts led to the acquisition of genomic alterations and the ability to disseminate^21^. In these two studies, hybrid tumours mimicked the genomic, histological and clinical features of human undifferentiated pleomorphic sarcomas, which are highly rearranged and aggressive tumours for which no specific genetic alteration and cellular origin has been identified^20,21^.

That observation led us to focus on the global involvement of cell fusion in sarcomagenesis. Sarcomas are rare aggressive tumours that derived from mesenchymal tissues^22^. They are subdivided in more 80 histotypes depending on their differentiation lineages and their molecular alterations^23^. Half of all sarcomas present no specific and recurrent genetic alteration and are characterized by major chromosomal reshuffling. This subgroup, called sarcomas with complex genetics, is composed of multiple histotypes, each one characterized by a specific differentiation lineage, but they all share a similar genomic complexity^22,24,25^. Knowledge is still sparse concerning the cell-of-origin in most of these tumours. Two models have been proposed: (a) the implication of mesenchymal stem cells that contribute to the development of different sarcomas following the engagement in a specific differentiation lineage; (b) the involvement of a near differentiated cells, which would explain the specific differentiation observed in tumours^26^.

In our previous studies, we showed that fibroblastic cell–cell fusion promotes the development of undifferentiated pleomorphic sarcoma, a histotype in which fibroblastic cells are thought to be the cell-of-origin^20,21^. We therefore hypothesize that cell–cell fusion can promote the development of different sarcoma histotypes according to the differentiation lineage of parent cells. To test this hypothesis, we isolated and fully characterized hybrids from fusions between transformed fibroblasts and immortalized myoblasts, i.e. cells which are thought to be the cell-of-origin of skeletal muscle sarcomas^27–30^.

## Results

### Hybrids inherit tumour initiation capacity and acquire metastatic properties

**Figure 1:**
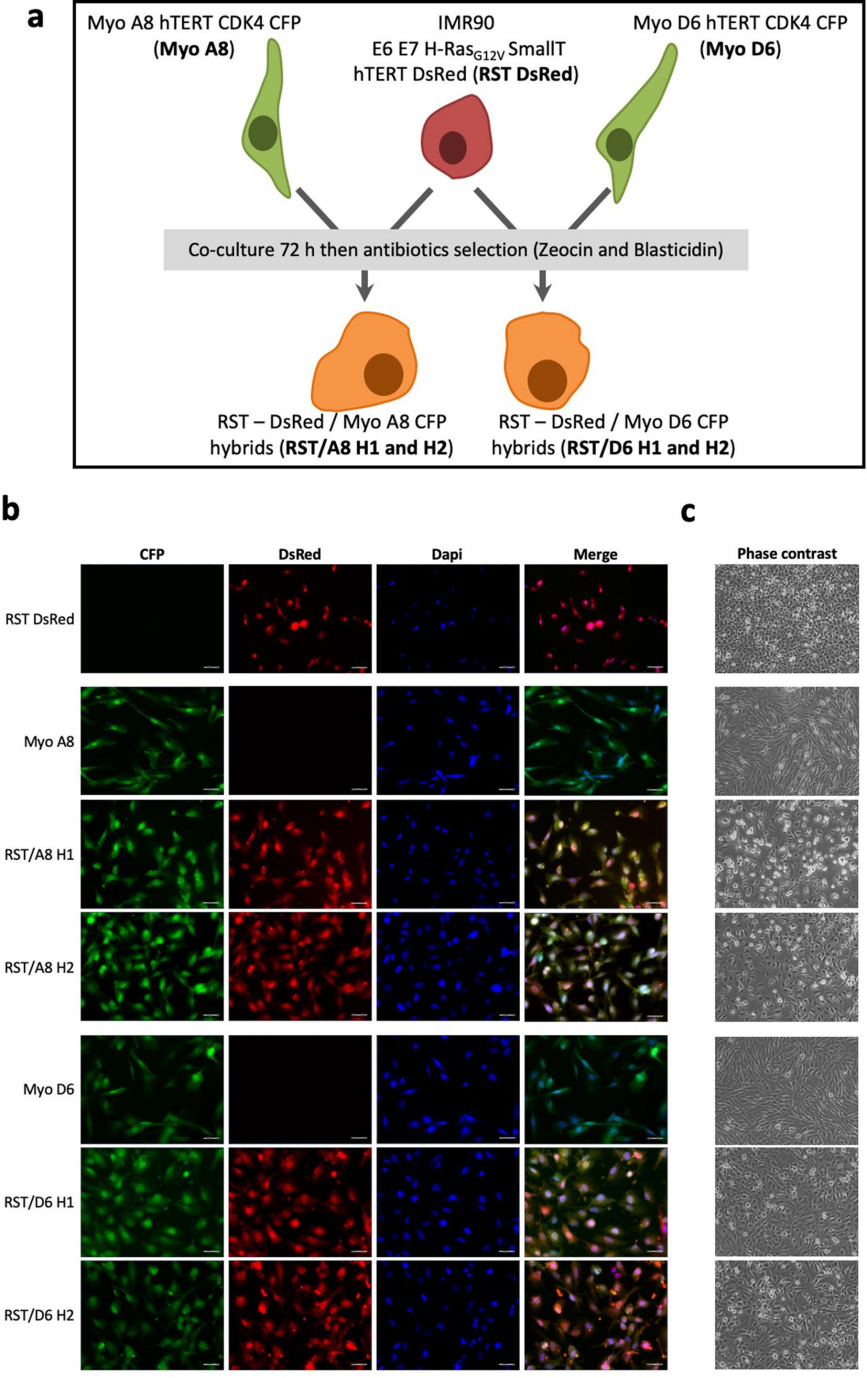
Hybrid selection and identification. (**a**) Schema of hybrid generation. RST-DsRed + is co-cultured with Myo A8 CFP + or Myo D6-CFP + cell lines and spontaneous fusion DsRed+–CFP+ cells are isolated. Hybrid cells are selected following antibiotics treatment (Zeocin and Blasticidin). (**b**) Fluorescence panels of parental RST DsRed, Myo A8 CFP and Myo D6 CFP and all hybrids cells. Scale bar = 50 μm. (**c**) Phase contrast panel of parental RST DsRed, Myo A8 CFP and Myo D6 CFP and all hybrids cells. (Objective = 5x).

Co-culture of a fibroblast transformed cell line, IMR90 E6E7 RST DsRed (RST), and an immortalized myoblast cell line (either Myo A8 CFP or Myo D6 CFP) were performed. After three days, spontaneous hybrids were isolated by retaining double antibiotic-resistant cells (Fig. 1a). All selected cells expressed both DsRed and CFP, thereby confirming that they were hybrid cells obtained from cell fusion (Fig. 1b). Although myoblasts are prone to the formation of multinucleated syncytia, these hybrids had one nucleus and corresponded to synkaryon (Fig. 1b,c).

**Figure 2:**
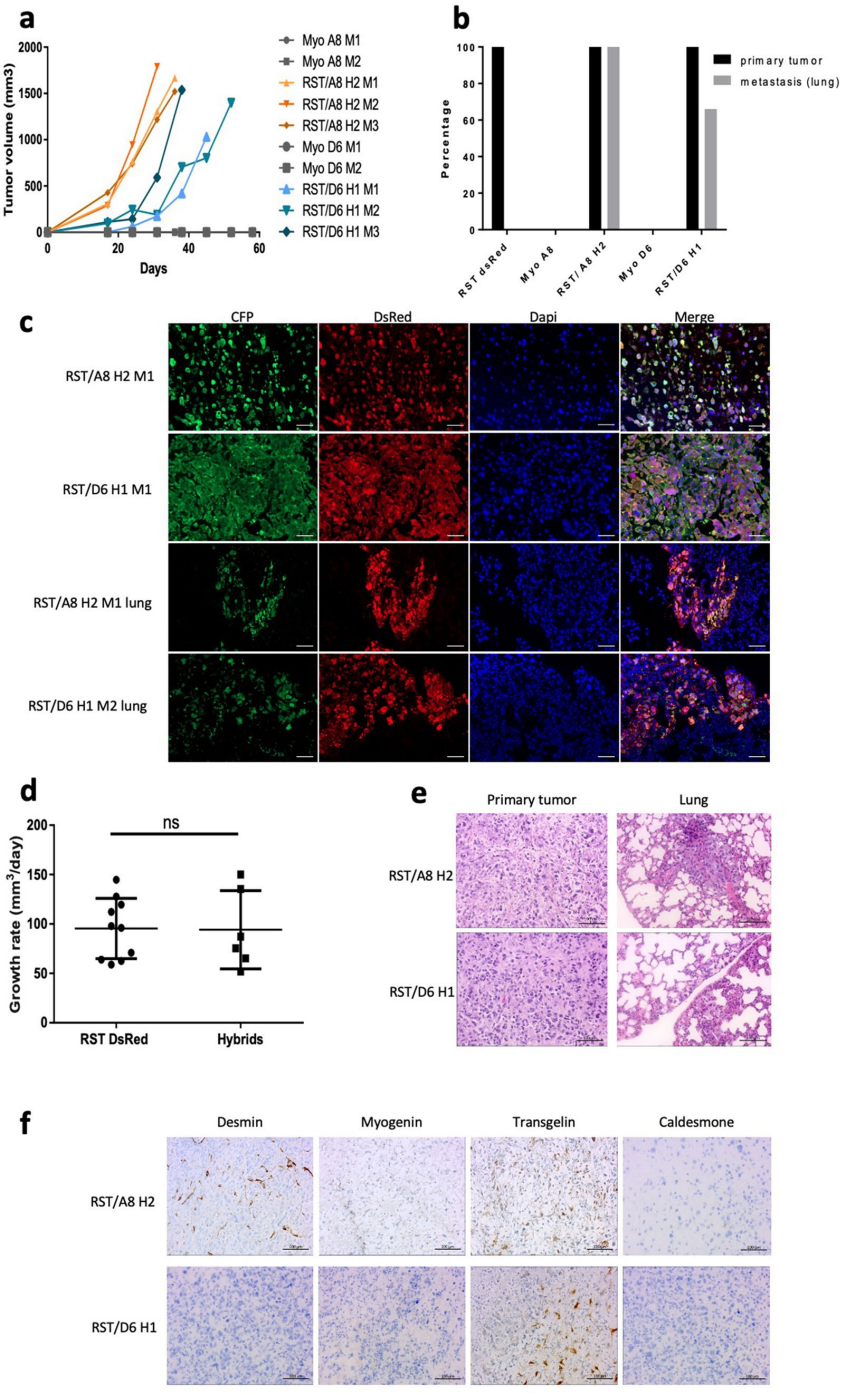
Hybids are more aggressive and lead to lung metastases. (**a**) Tumour growth curve of RST/A8 H2, RST/D6 H1 and myoblast cell lines after subcutaneous xenograft in NSG mice. Myoblast cells did not lead to tumour growth in mice, as indicating by overlapping grey lines. (**b**) Subcutaneous primary tumours (black bars) and secondary tumours (grey bars) incidence. (**c**) Immunofluorescence of hybrids tumours and lung metastasis. Scale bars = 50 μm. (**d**) Tumour growth rate after injection of RST DsRed (n = 10) and hybrids (n = 6). Ns: non-significant. (**e**) HE staining of primary tumours and lung metastases developed from RST/A8 H2 and RST/D6 H1. (**f**) Immunohistochemistry staining of desmin, myogenin, transgelin and caldesmone on primary tumours of hybrids. Scale bar = 50 μm.

RST DsRed injection promotes tumour formation in mice but not metastatic spread^20,21^. To establish whether hybrids had oncogenic properties, the parental myoblast cell lines, RST/A8 H2 (from fusion between RST DsRed and Myo A8 cell lines) and RST/D6 H1 (from fusion between RST DsRed and Myo D6 cell lines) were injected subcutaneously in mice. As expected, immortalized myoblast cell lines did not induce tumours in animals (Fig. 2a,b). In contrast, tumours were observed upon hybrids grafting after 17 days in 100% of animals, as RST DsRed (^20^ and Fig. 2a–c, Supplementary Fig. 1). Growth rates of hybrid tumours were similar to RST DsRed, indicating an inheritance of this phenotype in hybrids (Fig. 2d;^20^).

To test whether hybrids promoted metastatic dissemination, we looked for secondary tumours after sacrifice by measuring fluorescence in all lungs and organs showing an abnormal visual aspect. Metastases were identified only in mice with a hybrid tumour and were localized to the lungs in five out of six mice (83%). No second tumour site was identified in the parental condition, indicating the acquisition of the ability to disseminate through cell fusion by hybrids (Fig. 2b,c,e, Supplementary Fig. 2).

### Loss of myogenic markers and properties following cell fusion

Histological analysis of primary tumours from hybrids showed both a pleomorphic aspect and many mitotic cells (Fig. 2e). No expression of myogenin, a specific skeletal muscle marker, was observed in tumours, and desmin, a general muscle marker, was heterogeneously and exclusively expressed in RST/A8 H2 tumours (Fig. 2f). In contrast, transgelin, a smooth muscle marker, showed a heterogeneous expression in all hybrid tumours, while caldesmone was not detected in any tumour. Briefly, the incomplete expression of these muscle markers indicated that these tumours were undifferentiated pleomorphic sarcomas with incomplete muscle differentiation, in agreement with the WHO sarcoma classification^22^.

To test whether the non-expression of some myogenic markers in hybrid tumours was associated with a loss of muscle differentiation properties, all parent cells and hybrids were cultured in a specific differentiation medium. After seven days, multinucleated cells were observed in myoblast cell lines (Supplementary Fig. 3). No modification in cell shape was observed in RST DsRed. In contrast, hybrids formed aggregates after three days but no multinucleated myofiber was found. At seven days, hybrid cells were longer but contained one nucleus. The inability of hybrids to form syncytia also demonstrated a loss of muscle differentiation properties following cell fusion. This observation indicated a loss of myoblast properties by hybrids and a conservation of the transformed traits of RST-DsRed.

### Aggressiveness of hybrids comprises inherited dominant traits together with acquired new ones

**Figure 3:**
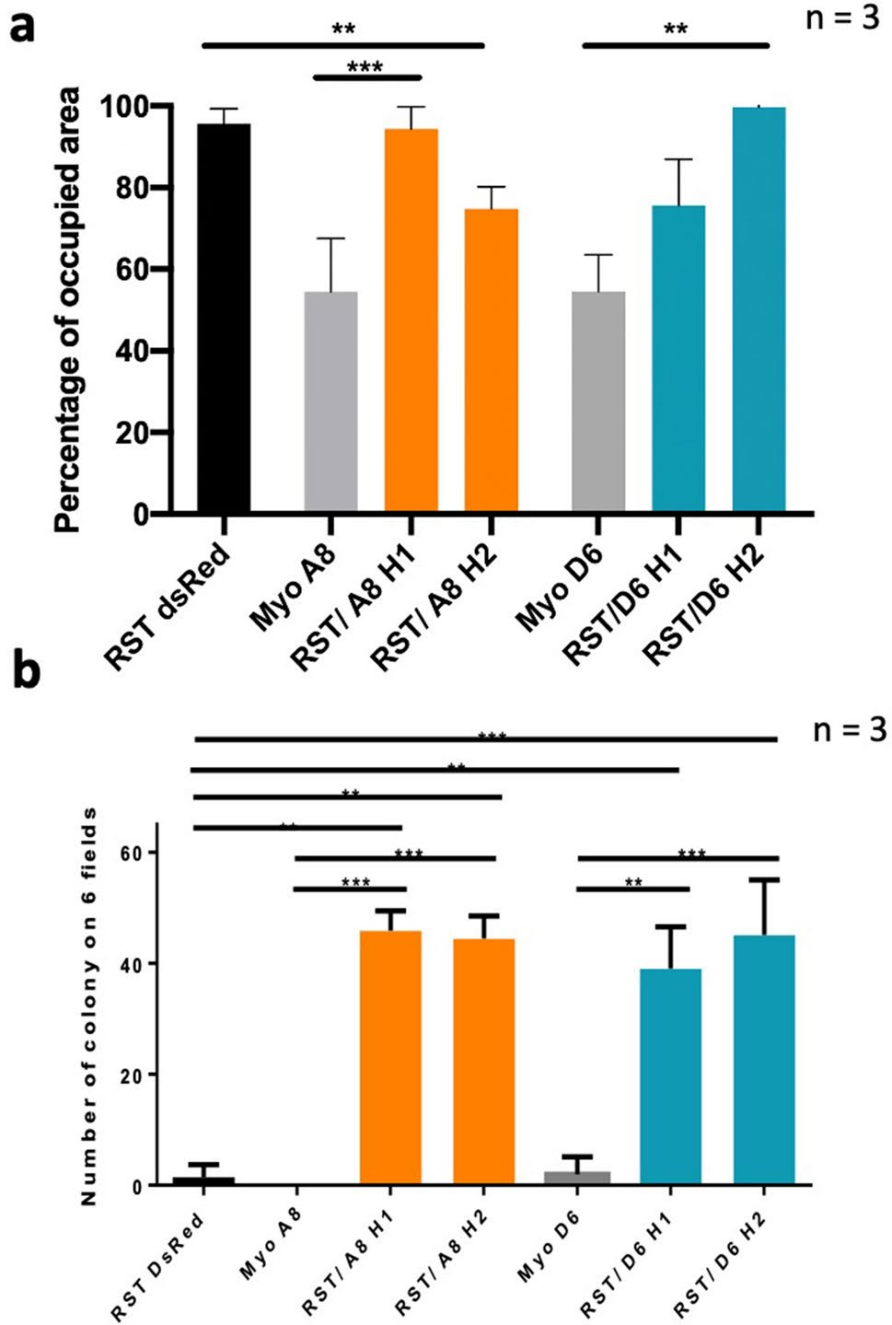
Hybrids are more aggressive than parental cell lines. (**a**) Evaluation of hybrid migration. Experiments performed in triplicate (n = 3). (**b**) Number of colonies obtained by soft agar assay. Data represent average number of colonies scored in a total of six fields (two fields per well, three wells per experiment, n = 3). Statistical analyses were done using Krustall Wallis’s test following Dunns’s multiple comparison test and Bonferroni’s adjustment (**P < 0.001, ***P < 0.0001).

To determine which new property induces the ability of hybrids to disseminate, we first tested their ability to migrate through in vitro assays. We found that hybrids show an intermediate migration ability compared to parental cells (*P* = 10^−3^; Fig. 3a). Although the ability to disseminate is mainly associated with this phenotype^31^, hybrids did not show an acquisition of mobility properties. However, other abilities are required during metastatic development, such as the ability to disseminate in the systemic circulation, seed and expand in a new microenvironment. Although these hybrids did not present acquisition of mobility, we hypothesized that they might be able to seed and expand.

Interestingly, a clonogenic assay demonstrated a significant difference between the hybrids and the parental cells with at least a 37-fold increase in the hybrids to form clones compared to the parental cells (Fig. 3b, Supplementary Fig. 4). While all hybrids had an equivalent clonogenic ability with a similar number of formed clones, RST/A8 H1 and RST/D6 H2 were able to form larger clones than those produced by RST/A8 H2 and RST/D6 H1, indicating that hybrids had variable phenotypes and that cell fusion led to different outcomes.

### Fusion leads to a genome reorganisation

**Figure 4:**
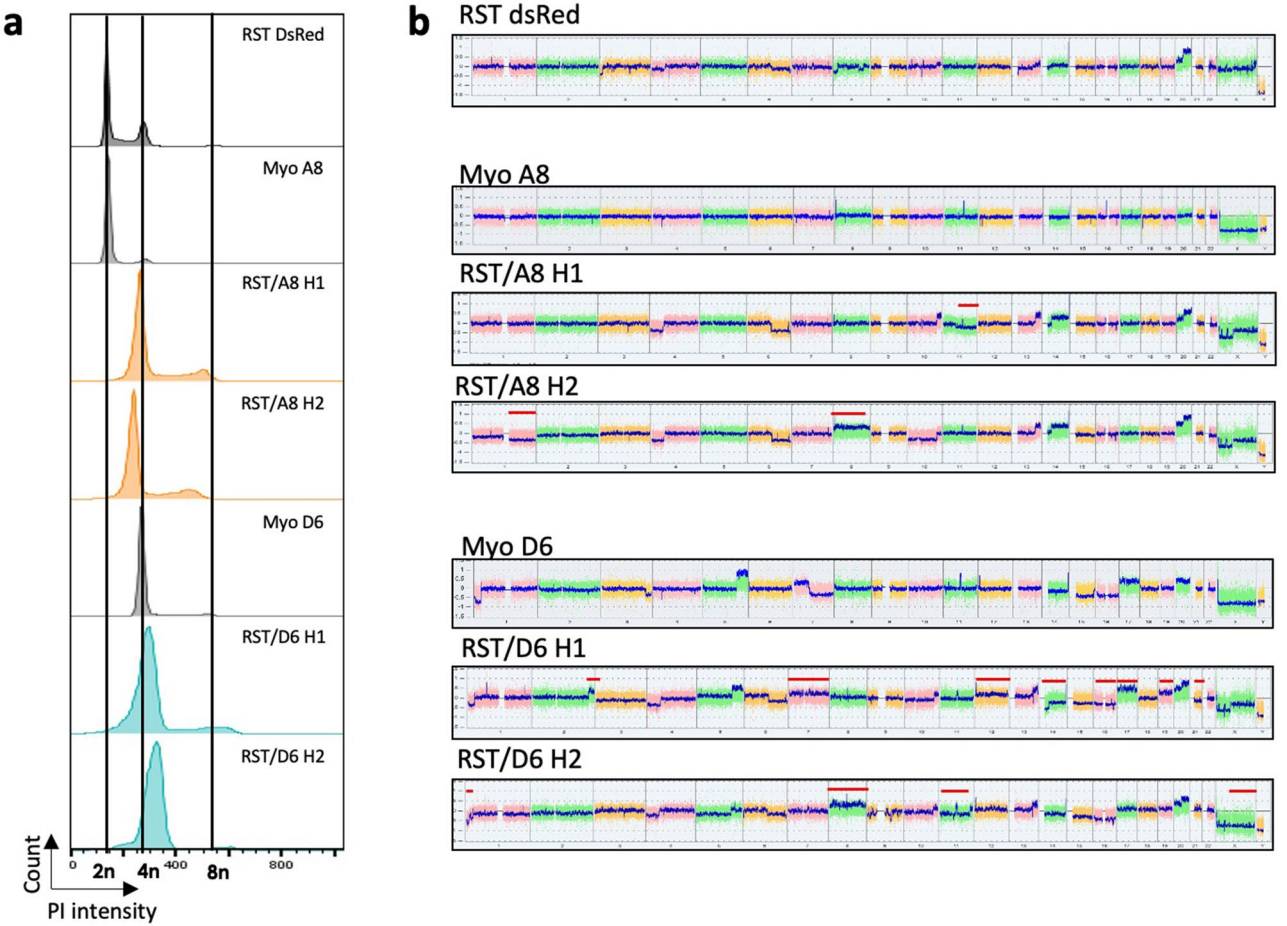
Hybrids have rearranged genome. (**a**) Cell cycle analysis by measurement of PI intensity for RST DsRed, Myo A8, Myo D6 and hybrids. RST/A8 hybrids which are all tetraploid as compared to diploid parental cells and RST/D6 hybrids are pentaploid as compared to diploid RST DsRed and tetraploid Myo D6 parental cells. Vertical lines indicate 2n, 4n and 8n ploidies. (**b**) Genomic profiling of RST DsRed, myoblast and hybrids cell lines analysed using the Chromosome Analysis Suite software v3.1. Copy number variations (CNVs) demonstrate that hybrid genome is rearranged upon fusion. x axis: chromosome 1 to chromosome Y; y axis: log2 ratio. Post-fusion new genomics alterations are indicated by red lines.

Proliferation upon cell fusion induces genomic merging and we previously described that fibroblast hybrids present copy number variations and new structural rearrangements upon fusion, demonstrating chromosomal instability^20,21^. Chromosome number analyses showed that 88 and 80 chromosomes were observed in RST/A8 H1 and RST/A8 H2, respectively (hybrids are pseudo-tetraploid) though diploid RST and Myo A8 have 46 and 48 chromosomes, respectively (Fig. 4a, Supplementary Fig. 5a,b). In RST/Myo D6 models, Myo D6 cells presented 94 chromosomes (4n) and hybrids had 112 and 115 chromosomes, their genomes presenting a 5n ploidy (Fig. 4a, Supplementary Fig. 5a,b). Hybrids of both models did not contain the sum of chromosomes of the respective parent cell lines, indicating a loss of genomic material (from some chromosomes to one entire set of chromosomes for RST/D6 hybrids) following cell fusion.

Genomic profiles of RST/A8 hybrids showed one new genetic alteration in RST/A8 H1 (loss of chromosome 11q arm) and two in RST/A8 H2 (loss of chromosome 1q arm and gain of chromosome 8; Fig. 4b). In the RST/D6 model, eight new genetic alterations were observed in RST/D6 H1 including five gains and four new genetic alterations in RST/D6 H2 including three losses (Fig. 4b). Many of these genetic alterations were sub-clonal in RST. Interestingly, the hybrids come from a specific subpopulation of RST, suggesting a selective advantage of this population to induce viable hybrids.

In the clonal evolution model, intra-tumour genetic instability induces acquisition of new genetic alterations and the emergence of clones with metastatic ability^32,33^. To test if hybrid metastatic spread is associated with new genetic alteration(s), we analysed the genomic profiles of hybrid primary tumours and compared them with the genomic profiles of their respective lung metastases. No modification was observed between the cell lines, primary tumours and metastases (Supplementary Fig. 6). Therefore, the ability of hybrids to disseminate is due neither to an acquisition of CNV following engraftment nor to the selection of a subclone with specific CNV.

Since none of the parental cell lines was able to metastasize, we analysed all punctual variants that were acquired and expressed following cell fusion or inherited by myoblast and were identified in RST/A8 H2 and RST/D6 H1 hybrids but not in RST DsRed. Eleven variants are identified, nine localized on 3′UTR regions but not in regions known to be targeted by miRNA. Among two other variants, one is synonymous and the other has already been described and is not associated with a clinical disorder (rs1620560). In the view of the above, no punctual variant demonstrated an association with a phenotype observed in the hybrids.

### Hybrids have a specific transcriptomic profile

Transcriptomic analysis showed that 610 and 1 195 genes are overexpressed and that 570 and 1 128 are down regulated in hybrids compared RST DsRed and myoblasts cell lines, respectively.

**Figure 5:**
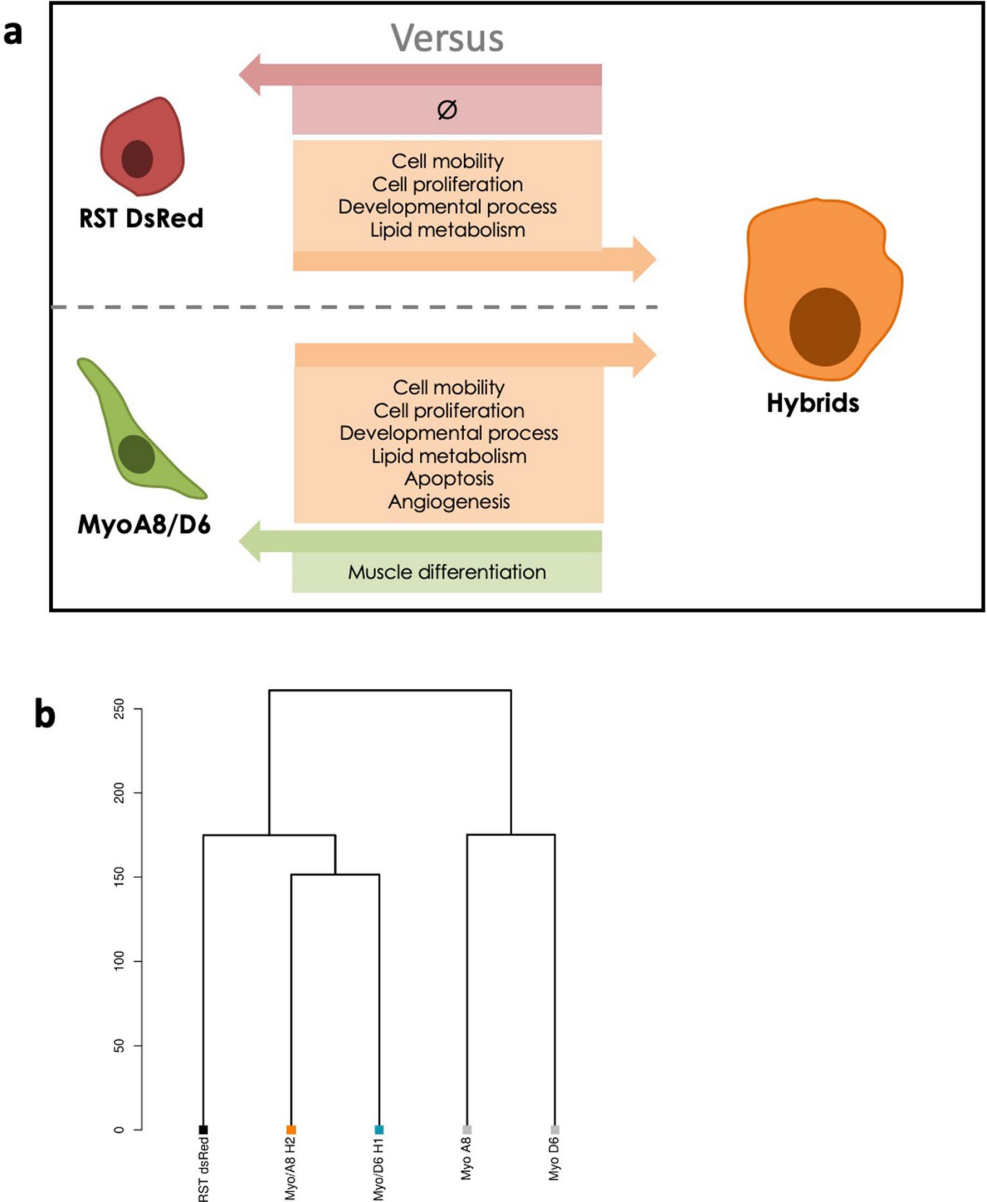
Hybrids presenttranscriptomic remodelling. (**a**) Schema of pathways overexpressed in parental cells compared to hybrids and in hybrids compared to parental cells. Each arrow indicates enriched Gene Ontologies in different cellular entities. On the top, pathways enriched in hybrids compared to RST DsRed are indicated in orange square. No pathway is enriched in RST DsRed compared to hybrids as indicated in the red square. On the bottom, pathways enriched in hybrids compared to myoblast cells are showed in orange square. Muscular differentiation pathway enriched in myoblasts compared to hybrids is indicated in green square. (**b**) Transcriptomic profile clustering of parental and hybrid cell lines.

Gene Ontology demonstrated that myoblast cell lines overexpressed genes involved in muscle differentiation pathways (*P* < 10^−4^) compared to hybrids in agreement with in vitro assays. Among 570 genes overexpressed in RST DsRed compared to hybrids, no pathway was identified as specifically enriched. (Fig. 5a). Interestingly, GO analysis on 610 and 1 195 genes overexpressed only in hybrids compared to myoblasts or RST DsRed parental cell lines respectively showed similar results. Both gene sets were involved in similar processes: developmental process and morphogenesis (*P* < 10^−4^), cell mobility (*P* < 10^−5^), cell proliferation (*P* < 10^−4^), and lipid metabolism (*P* < 10^−6^; Fig. 5a). Pathways involved in angiogenesis (*P* > 10^−3^) and apoptosis (*P* > 10^−3^) were enriched in hybrids compared to myoblast cell lines only. Finally, clustering analysis revealed that the hybrids presented similar transcriptomic profiles and were closer to RST than myoblasts, in agreement with phenotypic data (Fig. 5b).

## Discussion

The contribution of cell fusion to tumour cell dissemination has been well documented, beginning with the first hypothesis of Otto Aichel a century ago until more recent studies describing hybrids in metastatic patients^6,7,12–15,20,21^. We previously reported that cell fusion is a one-step evolutionary event, leading to the global reshuffling of hybrid genome, promoting tumour evolution and mimicking the development of undifferentiated pleomorphic sarcomas^20,21^. This present study confirms and adds to our previous results, demonstrating that cell-fusion can contribute to the diversity of differentiation lineages observed in sarcomas but also favour the emergence of new ability like metastatic traits.

The most widely reported tumorigenic consequence of cell fusion is the emergence of motile tumour cells following fusion between cancer cells and leucocytes^6,7,11,13,14^. All these studies described cell fusion as a process in which parental properties are transferred to hybrid cells. We recently reported that cell fusion between immortalized and transformed fibroblasts induces the formation of metastatic hybrids following the acquisition of migration ability by fused cells^21^. To our knowledge, this was the first time that the acquisition of the ability to disseminate following cell fusion had been described. The present study confirms that only hybrids can metastasise and this trait is associated with the acquisition of clonogenic capacity. Contrarily to our previous work, hybrids did not show an increase of in-vitro mobility compared to transformed parental cell line, confirming that dissemination ability is not exclusively related to this property. Although mechanisms related to metastasis outcome are challenging to decipher, notably regarding features required for dissemination, it seems in this model that the new ability to seed and to proliferate from a single cell acquired by fused cells governs the dissemination phenotype observed in hybrid tumours. Metastatic clones apparently emerge in different ways. Cell fusion contributes to the emergence of hybrids with specific phenotypes as a result of the inheritance or acquisition of properties, but also to the same purpose: promoting metastatic cells. Therefore, this cellular event contributes to tumour evolution by promoting heterogeneity and the emergence of aggressive subclones.

In the present work, cell–cell fusion induces large genetic remodelling and acquisition of a specific transcriptomic program. Among all the genes overexpressed, various gene sets were identified, mainly those involved in cell mobility, proliferation and angiogenesis, which is in agreement with the phenotypic properties of hybrids. We also observed overexpression of pathways involved in lipid metabolism. Deregulation of lipid metabolism through oncogenesis contributes to several cellular processes like migration and proliferation, as observed in hybrid cells^34^. Hybrids undergo extensive transcriptomic modifications, with the expression of pathways involved in tumour initiation, such as proliferation and tumour dissemination via an angiogenesis pathway and cell mobility.

Interestingly some gene sets involved in development process and morphogenesis were identified in hybrids, suggesting that hybrids acquire the properties of stem cell. According to the cancer stem cell (CSC) evolution model, tumours have an organization similar to that of normal tissue and are initiated from a pool of specialized cancer cells with stemness proprieties. Interestingly, one of the properties of CSC is to allow tumours to develop from a single cell^32^, a phenomenon observed only in these hybrids forming secondary tumours. This transcriptomic identity is thus in accordance with the clonogenic property and metastatic features characterizing exclusively hybrid cells. We therefore suggest that the metastatic ability of hybrids results from the stemness properties that they acquire.

This new transcriptomic profile is acquired in hybrid, while it is not observed in parental cells, suggesting that cell fusion may contribute to the emergence of CSC. Three mechanisms are proposed to induce CSC: accumulation of genetic alterations in stem cells driving oncogenesis, dedifferentiation of cancer cells, or the fusion between differentiated cells and stem cells^5,32^. In the present study, stemness properties were likely conferred by the fusion of two non-stem cells, suggesting a novel mechanism not reported until now.

Cell–cell fusion is known to promote genetic alterations from the first single cell to all successive daughter cells^18,20,35,36^. It contributes to phylogenetic evolution in plants where allopolyploidy (fusion between different species) leads to “genomic and epigenomic chaos” via the genetic and epigenetics divergence of both original cells^37^. We hypothesize that the fusion of myoblast and fibroblast cells promotes this chaos and leads to the fusion of genetically and transcriptomically divergent cells. Hybrids might acquire new genetic alterations, which could contribute to the aggressive properties observed in many tumour types and generate epigenetic modification, leading to the specific transcriptomic program identified in hybrid^21,38–40^.

Identifying the mechanism(s) involved in tumour dissemination is a major challenge to depict the most accurate portrait of tumour evolution. While only a fraction of cancer cells are known to have metastatic properties, it is unclear whether the emergence of the metastatic initiating cancer cells is the consequence of genetic modification, as in the clonal evolution model, or whether they arise from a pool of specialized cancer cells with stemness properties^32,33,41,42^. Although the identification of the appropriate model for each cancer type is sometimes difficult, those are not necessarily exclusive so there is a need for identifying cancer cells capable of metastatic properties. Since cancer evolution is determined by genetic and/or phenotypic evolution, cell–cell fusion is a promising mechanism in which hybrids harbour profound changes in genome organisation and phenotype compared to parental cells.

Our previous work showed that cell fusion promotes genomic reshuffling, either by establishing unstable genetic hybrids during tumour initiation or by massive but then stable genomic reshuffling during tumour progression^20,21^. This present study confirms previous data showed in^21^. Cell–cell fusion between transformed and immortalized cells induces large genetic remodelling without contributing to progressive genetic instability. No genetic alteration is acquired during in-vivo tumour growth. Following the clonal evolution model which posits that new properties are acquired through genetic variation, ability of hybrids to disseminate would be acquired before in-vivo engraftment and would be the direct consequence of cell–cell fusion. In addition, the tolerance to massive genetic reshuffling and genetic instability would vary from one hybrid to another, probably according to whether these properties are present or not in the parent cells.

The histotype of hybrid tumours is influenced by the differentiation lineage of parent cells, hybrid cells inherit properties from parental cells thus contributing to the specific classification of tumour cells. RST/Myo hybrids express desmin and transgelin, so the resulting tumours are classified as undifferentiated pleomorphic sarcomas with an incomplete muscular differentiation. However, fused cells do not inherit from the capacity to form myotube and do not conserve a myoblast expression program. Although cell fusion is widely considered as a horizontal transmission process where the hybrid phenotype results from the union of parental phenotypes, the regulation of parental properties after cell fusion is likely more complex. Following this fusion event, properties of transformed RST DsRed would be dominant compared to differentiation phenotype of myoblast, this can be explained here by the tumorigenic phenotype induced by oncogenes of RST DsRed. However, this cannot explain the loss of myoblast markers since the transformation of myoblasts by similar oncogenes promoted tumour development in mice expressing skeletal muscle markers like desmin and myogenin^28^. The loss of parental markers is consequently observed after the fusion event. Instead, it has been reported that a heterokaryon between a cancer cell line and a myoblast lost muscle properties with the loss of MyoD expression^43–46^. It seems that hybrids do not possess all the properties of the parental cells but might select and combine the advantageous ones.

We previously reported that fusion between two fibroblasts led to hybrids classified as undifferentiated pleomorphic sarcomas^20^. We now described that cell–cell fusion as a mechanism inducing the formation of undifferentiated pleomorphic sarcomas with an incomplete muscular differentiation when it involves myoblasts. While cellular origin of pleomorphic sarcomas has not yet been discovered, cell fusion could be a major evolutionary process, that accounts for genomic reshuffling and contributes to the specific differentiation lineage observed in pleomorphic sarcomas. Thanks to the acquisition of genomic alterations and the phenotypic properties of parent cells, cell fusion might be one of the very first cellular event of sarcoma inception.

## Materials and methods

### Cell line

IMR90 fibroblasts, myoblasts A8 and D6 cell lines and hybrids were cultivated in growth medium composed of 60% of DMEM (Thermofisher Scientific, Waltham, Massachusetts, USA), 20% of M199 medium (Thermofisher Scientific), 20% of decomplemented foetal bovine serum, 1% of penicillin–streptomycin and dexametazone at 37 °C in a humidified incubator CO_2_ incubator. Fibroblasts cell line were kindly provided by Martin Teichmann (Inserm U1212, Bordeaux, France) and myoblast cell lines were provided by Bénédicte Chazaud (Inserm U1217, Lyon, France).

IMR90 cell line is a transformed cell line, hosting the two papilloma viruses E6 and E7 (targeting p53 and pRB respectively), HRAS_G12V_, SV40 small T and hTERT. It is transformed according to the model described by^47,48^. Myoblasts were immortalized cell lines hosting hTERT and CDK4.

### Hybrid selection

IMR90 was infected with pLenti-Puro with DsRed and pSlik-Zeo vectors and myoblasts with pLenti-Blast with CFP vector (generous gift of Dr. Richard Iggo). A co-culture of 75,000 cells of each parent cell lines was performed in six well plates. Spontaneous hybrid cells were observed after 72 h of co-culture and were selected by adding antibiotics (250 μg/ml of zeocin and 18 µg/ml of blasticidin, Thermofisher Scientific).

### DNA extraction and DNA-array

DNA extraction and DNA-array were realized as described^20^. Briefly, genomic DNA was extracted using the standard phenol–chloroform method and quantified using the NanoDrop ND-1000 Spectrophotometer (Thermofisher Scientific). Genomic profiling was performed using the Affymetrix CytoScan HD Arrays (Thermofisher Scientific) for all cell lines and mice tumours according to the manufacturer’s instructions. CEL files obtained by scanning the arrays were analysed using the Chromosome Analysis Suite software v3.1 (Thermofisher Scientific; http://www.affymetrix.com/support/technical/byproduct.affx?product=chas) and the annotations of the genome version GRCH37 (hg19).

### Cell cycle assay

Cell cycle assay was realized as described^20^.

### Chromosome spread

Chromosome spread was realized as described^20^.

### RNA sequencing and bioinformatics tools

RNA extraction and sample preparation were performed as described^49^. RNA sequencing was performed using stranded strategy. Bioinformatics analyses were performed as previously described (bioinformatics pipelines for gene expression^50^).

From curated BAM files, SAMtools and BCFtools (v0.1.19) detected mismatches at any position with a minimum depth of five reads where alternative bases were above 30%^51,52^. Then, all mismatches were investigated in all samples (possibly below 30% of alternative bases).

Variants were then annotated with the different databases using ANNOVAR (v2017/07;^53^. We excluded known variants in the 1000 Genomes Project or in the dbSNP, non-exonic or synonymous variants.

Differential gene expression (DGE) was performed on raw counts, then normalized and analysed using DESeq2 (v1.16.1) from Bioconductor^54^. Clustering methods were performed using cluster (v2.07). Dendograms were produced by Ward’s agglomerative method with Euclidean distance^55^. All these methods were applied on log2(FPKM + 1) expression values.

Gene ontologies were computed using Gorilla considered significant below false-discovery rate (FDR) *Q* < 0.05^56^.

Miscellaneous computations, filters, statistics and plots were performed with R (3.4.4).

### Tissue immunofluorescence

Tissue immunofluorescence was realized as described^20^. Briefly, 4 µm paraffin-embedded tissue section placed on a glass slide was de-paraffinized in three baths of xylene for 5 min and rehydrated in a series of ethanol baths. For antigen retrieval, slides were incubated in DAKO Target Retrieval Solution, pH6 (DAKO, Carpinteria, California, USA), for 20 min in a microwave oven. Slides were incubated with mouse anti-CFP (Santa Cruz Biotechnology, Dallas, Texas, USA, sc9996, dilution 1/50) and rabbit anti-RFP (Abcam, Cambridge, UK, ab62341, dilution 1/50) primary antibodies for 1 h at RT after proteolytic epitope retrieval in citrate buffer (pH 6.0). Then, Alexa Fluor 488 goat anti-mouse (Molecular Probes, Eugene, Oregon, USA, A11001, dilution 1/400) and 647 goat anti-rabbit (Molecular Probes, A21245, dilution 1:400) secondary antibodies were incubated for 1 h at RT. Slides were mounted using the Vectashield mounting medium plus DAPI (Vector Laboratories). Images were acquired on a Zeiss Cell Observer microscope (Zeiss, Oberkochen, Germany).

### Immunohistochemistry and H&E staining

Four µm-thick serial sections were cut from the formalin-fixed paraffin-embedded (FFPE) tumour blocks and were used for haematoxylin and eosin (HE) staining and immunohistochemical analysis. HE staining was performed according to standard protocols.

Slides of tumour were de-paraffinized in xylene, hydrated in alcohol and baked in a microwave. Endogenous peroxidase was blocked. Immunohistochemical analysis was performed in all cases with the following antibodies: desmin (clone D33, Dako), myogenin (clone LO26, Novacastra), transgelin (clone D33, LSBio, Seattle, WA, United States) and caldesmone (clone h-CD, Dako). Appropriate positive and negative controls for each antibody were included. Pictures were captured using a Zeiss Cell Observer Microscope (Zeiss).

### Crystal violet clonogenic assay

To evaluate the ability to produce a progeny from a unique cell, 1000 cells were seeded in six-wells plates and cultured for 10 days. Colonies were fixed with 70% ethanol for 5 min and stained with 2.3% crystal violet solution (Sigma-Aldrich) for 5 min. The number of colonies was scored after 10 days by manual counting using an Olympus CKX41 Microscope and a 4 × objective (Olympus, Tokyo, Japan).

### Migration assay

For the wound healing assay, 4 × 10^5^ cells were plated onto a six-wells plate. Twenty-four hours later, a strip of cells was removed from the monolayer of cells using a pipette tip. Phase contrast images were acquired with a 10 × objective at the time of the scratch and 24 h later using an Olympus CKX41 Microscope (Olympus).

### Myotubes differentiation assay

Cells were seeded at confluence in medium composed of DMEM (Thermofisher Scientific), insulin (Sigma Aldrich, I2643, 1 mg/ml) and transferrin (Sigma Aldrich, T8158, 50 mg/ml) for seven days. Images were acquired with an Olympus CKX41 Microscope and a 4 × objective (Olympus).

After seven days of differentiation, cells were fixed with paraformaldehyde 4%, permeabilized with Triton 0.5% solution and mounted using Vectashield medium plus DAPI (Vector laboratories) Images were acquired with a Confocal Zeiss LSM 510 microscope (Zeiss).

### In-vivo experimentations

In-vivo experimentations were realized as described^20^. Animals were maintained under specific pathogen-free conditions in the animal facility of Bordeaux University. Experiments were performed in conformity with the rules of the Institutional Animal Care and Use committee and submitted to the French Ministry of Education and Research (approval number DIR13109 and DAP-APAFiS-201802161802878) and all efforts were made to minimize animal suffering. For all experiments, 10^5^ cells were subcutaneous injected into the right dorsal flank of 6–8 weeks-old NOD.Cg-Prkdcscid Il2rgtm1Wjl/SzJ (NSG) mice. Tumours were measured twice a week using a calliper and volume tumour was calculated using the formula: V = length × width^2^/2. At the end of the experiment, mice were sacrificed by cervical dislocation. Tumours were then weighed and divided in three parts for cell culture, formalin fixation and nitrogen freezing. Growth rate was calculated with the segmental linear regression of GraphPad Prism (GraphPad Software, San Diego, California, USA). Statistical analyses were done using the Kruskal–Wallis test.

## Supplementary information

Supplementary Figures.

## Data Availability

The raw sequencing data is available from SRA, accession number PRJNA674043.
