# Supplementary material for: Cell–cell fusion of mesenchymal cells with distinct differentiations triggers genomic and transcriptomic remodelling toward tumour aggressiveness
Source: Sci Rep. 2020 Dec 10;10:21634. doi: 10.1038/s41598-020-78502-z (PMC7729932; doi:10.1038/s41598-020-78502-z)
Supplement: Supplementary file 1 — Supplementary Figures. [file 41598_2020_78502_MOESM1_ESM.pdf]

Supplementary information

Cell-cell fusion of mesenchymal cells with distinct differentiations triggers genomic and transcriptomic remodelling toward tumour aggressiveness

Lucile Delespaul, Caroline Gélabert, Tom Lesluyes, Sophie Le Guellec, Gaëlle Pérot, Laura Leroy, Jessica Baud, Candice Merle, Lydia Lartigue & Frédéric Chibon

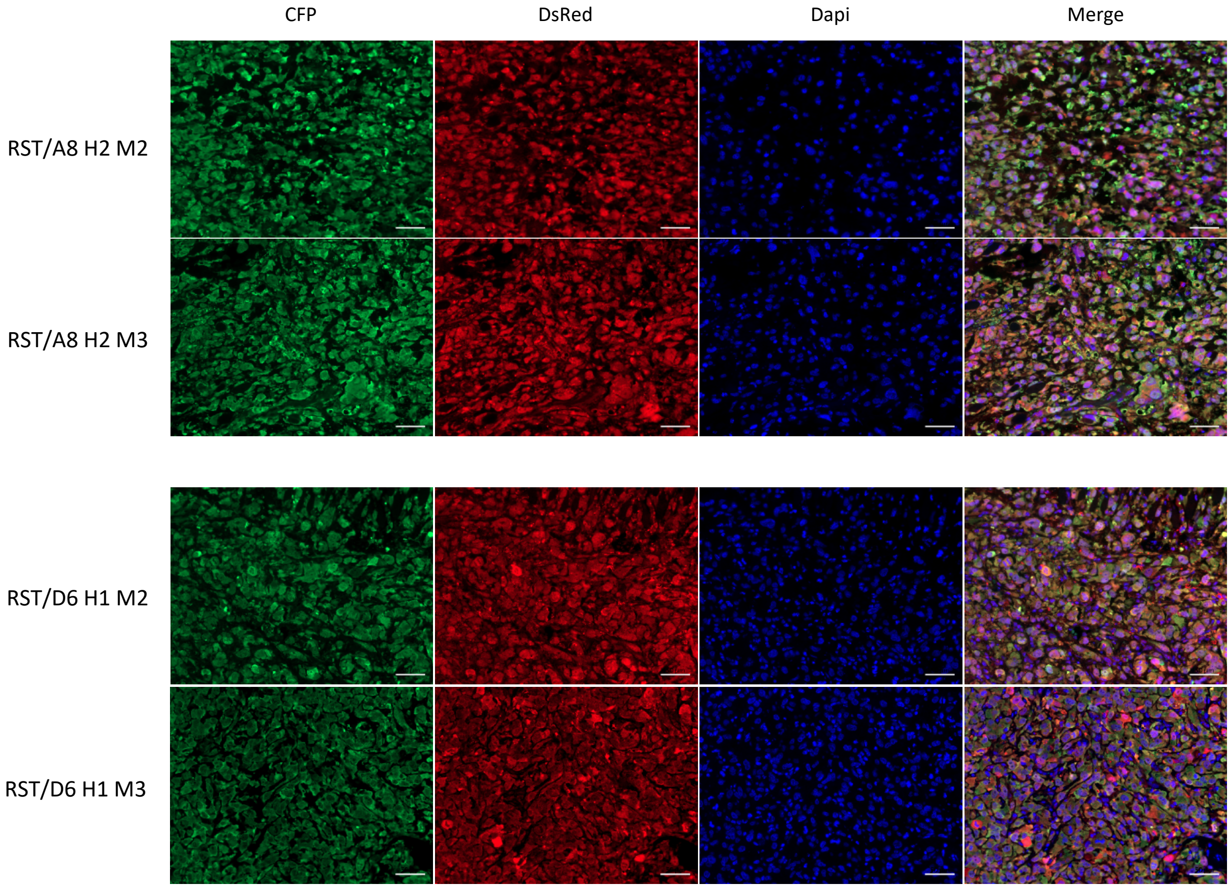

Supplementary Figure 1: Immunofluorescence of hybrids tumours. Scale bars = 50 µm.

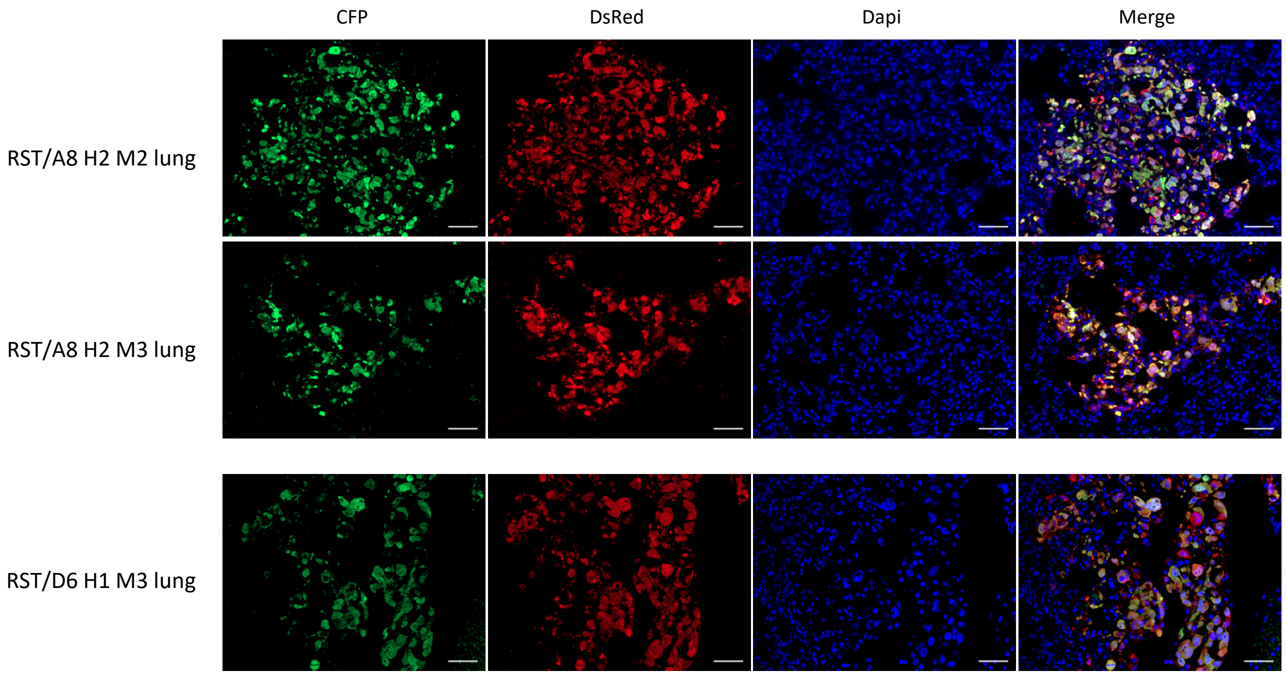

Supplementary Figure 2: Immunofluorescence of hybrids lung metastases. Scale bars = 50 μm.

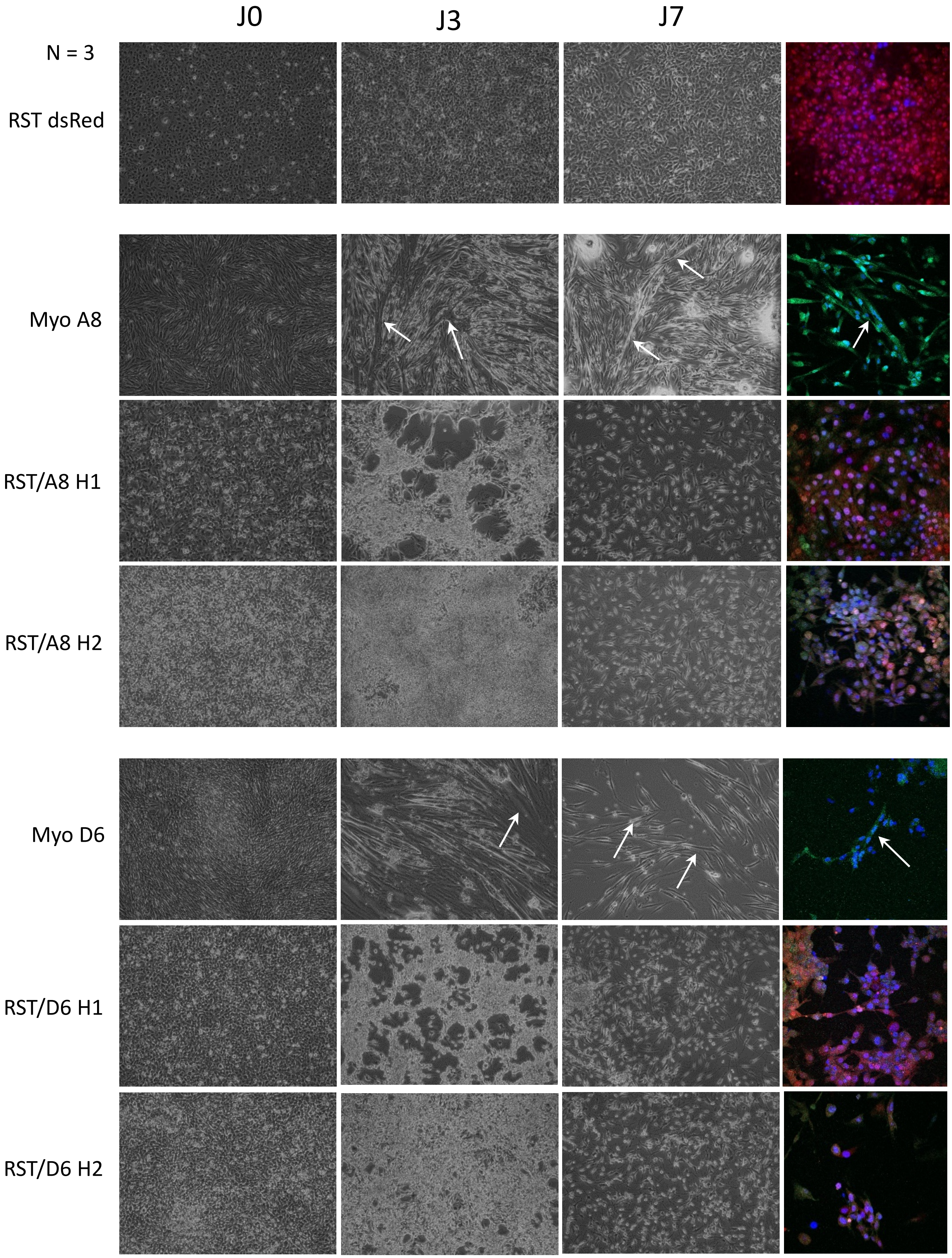

Supplementary Figure 3: Hybrids do not conserve myotube differentiation ability. Each cell line was cultured in differentiation medium for seven days and observed at day 0, 3 and 7. After 7 days, cells were fixed, nuclei were marked with Dapi (blue) and observed under confocal microscope. Myotubes are indicated with white arrows.

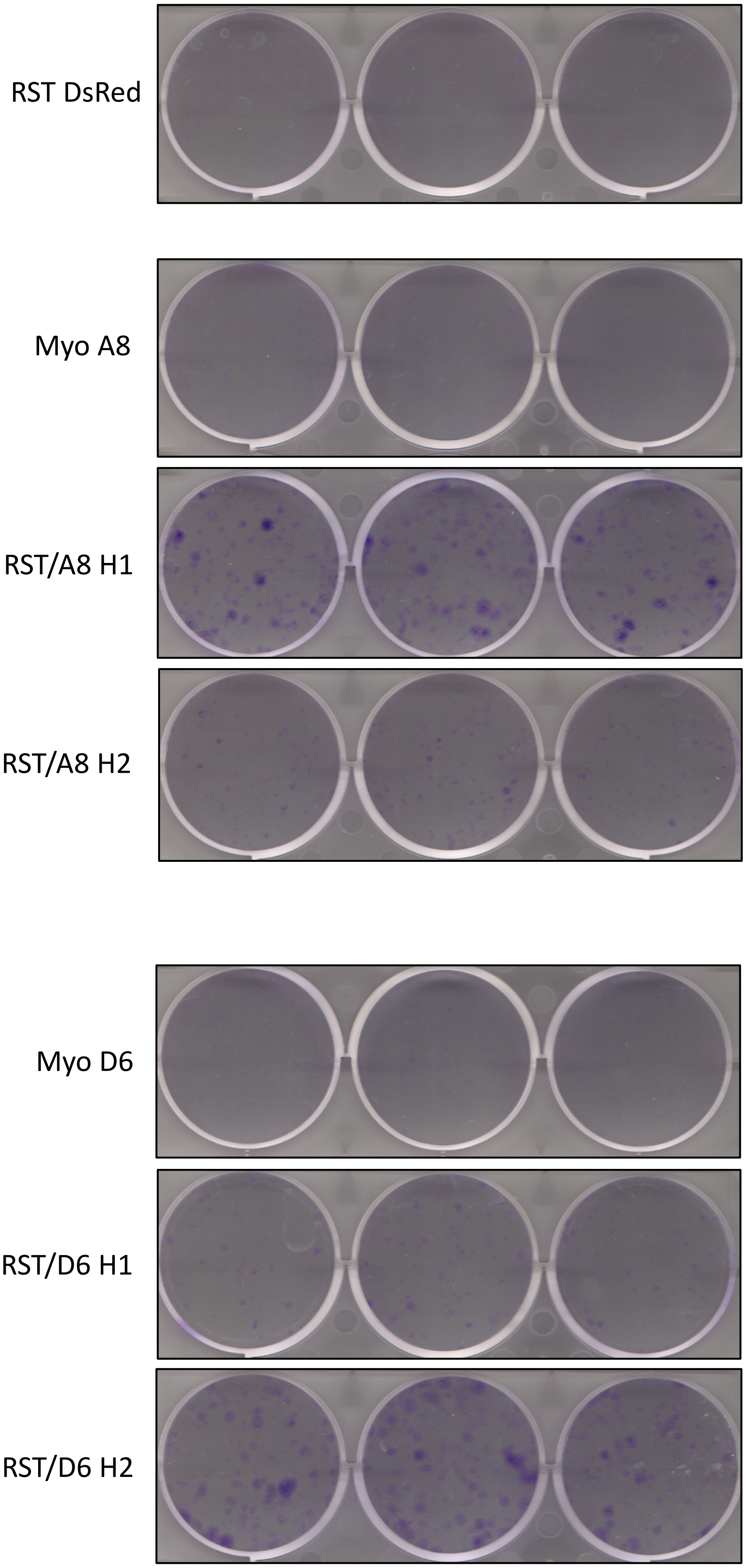

Supplementary Figure 4: Hybrids acquire clonogenic ability compared with parent cells. Colonies are marked with crystal violet. Experiment performed three times in triplicate.

A

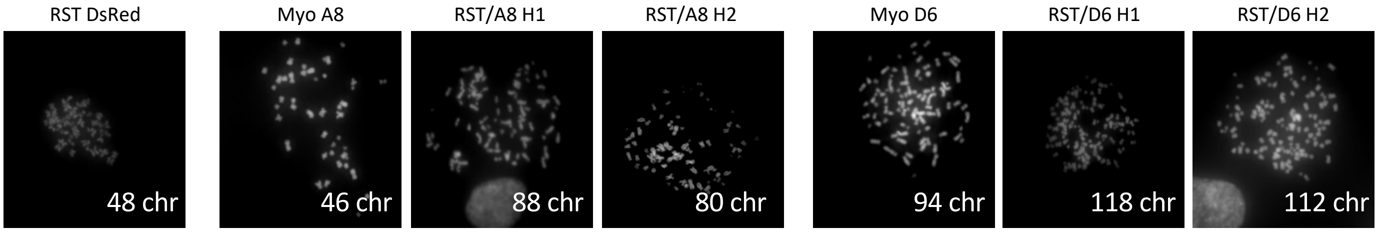

B

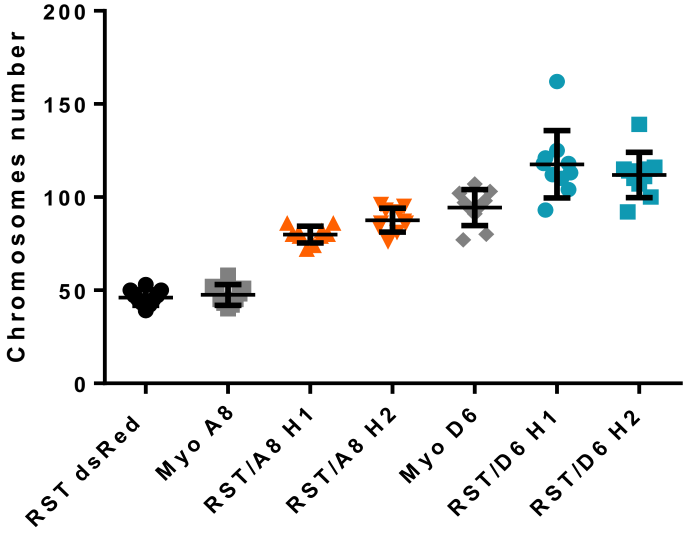

Supplementary Figure 5: Chromosome number analysis A). Chromosome spreads for each cell lines were performed and mean chromosome number is indicated (n=15). B) Average number of chromosomes of each cell line. Count performed on 15 metaphases. Mean and SD (black lines) are indicated.

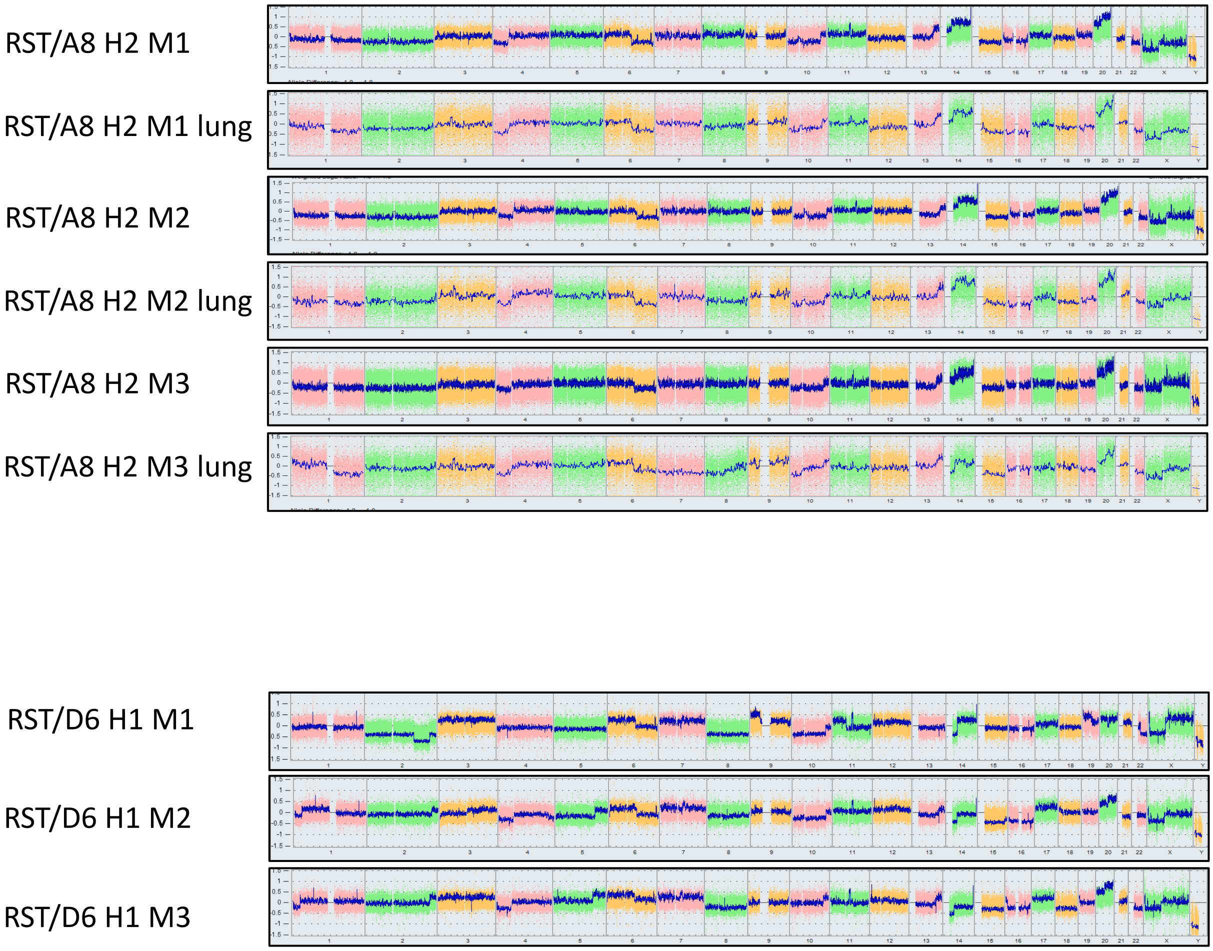

Supplementary Figure 6: Genomic profiling of hybrids tumours and associated metastases. Copy number variations (CNVs) demonstrate that hybrid genome is rearranged upon fusion. x axis: chromosome 1 to chromosome Y; y axis: log2 ratio.
